# Potassium Channels as Therapeutic Targets in Pulmonary Arterial Hypertension

**DOI:** 10.3390/biom12101341

**Published:** 2022-09-22

**Authors:** Gabriel Redel-Traub, Kevin J. Sampson, Robert S. Kass, Michael S. Bohnen

**Affiliations:** 1 Department of Medicine, Columbia University Irving Medical Center, New York, NY 10032, USA; 2 Department of Molecular Pharmacology and Therapeutics, Columbia University Irving Medical Center, New York, NY 10032, USA; 3 Division of Cardiology, Department of Medicine, Columbia University Irving Medical Center, New York, NY 10032, USA

**Keywords:** pulmonary hypertension, ion channel, channelopathy, KCNK3, ABCC8, pharmacology

## Abstract

Pulmonary arterial hypertension (PAH) is a devastating disease with high morbidity and mortality. Deleterious remodeling in the pulmonary arterial system leads to irreversible arterial constriction and elevated pulmonary arterial pressures, right heart failure, and eventually death. The difficulty in treating PAH stems in part from the complex nature of disease pathogenesis, with several signaling compounds known to be involved (e.g., endothelin-1, prostacyclins) which are indeed targets of PAH therapy. Over the last decade, potassium channelopathies were established as novel causes of PAH. More specifically, loss-of-function mutations in the *KCNK3* gene that encodes the two-pore-domain potassium channel KCNK3 (or TASK-1) and loss-of-function mutations in the *ABCC8* gene that encodes a key subunit, SUR1, of the ATP-sensitive potassium channel (KATP) were established as the first two potassium channelopathies in human cohorts with pulmonary arterial hypertension. Moreover, voltage-gated potassium channels (Kv) represent a third family of potassium channels with genetic changes observed in association with PAH. While other ion channel genes have since been reported in association with PAH, this review focuses on KCNK3, KATP, and Kv potassium channels as promising therapeutic targets in PAH, with recent experimental pharmacologic discoveries significantly advancing the field.

## 1. Introduction

Pulmonary hypertension (PH) encompasses a broad variety of diseases that differ in etiology, pathophysiology, therapeutic approach, and prognosis. The current clinical classification identifies five groups of PH. World Health Organization (WHO) Group 1 PH, known as pulmonary arterial hypertension (PAH), is a form of isolated pre-capillary PH. Isolated pre-capillary PH is defined hemodynamically by a mean pulmonary artery pressure >20 mmHg, a pulmonary artery wedge pressure ≤15 mmHg, and a pulmonary vascular resistance (PVR) >2 Wood units [1,2].

PAH is a severe cardiopulmonary disease classified by vascular remodeling and subsequent obstruction of distal pulmonary arteries leading to increased PVR, right-sided heart failure, and eventually death. PAH has been subcategorized by underlying etiology, including: idiopathic PAH; heritable PAH; drug- and toxin-induced PAH; PAH associated with HIV, connective tissue disorders, portal hypertension, congenital heart disease, PAH associated with schistosomiasis; and persistent PH of the newborn [1,3,4].

While the pathophysiology underlying PAH is recognized to be multifactorial, it is generally accepted that dysregulated proliferation and dysfunction of pulmonary arterial endothelial cells (PAECs) and pulmonary arterial smooth muscle cells (PASMCs) are major drivers in the development of PAH [5,6]. Genetic defects, epigenetic insults, metabolic disarrangement, hypoxia, shear stress, and/or inflammation create a milieu in which PAECs overproduce vasoconstrictive and mitogen molecules, such as endothelin-1 (ET-1), serotonin (5-HT), and thromboxane A2 (TXA), leading to PASMCs resisting apoptosis [6,7,8,9,10].

Research into hereditary causes of PAH has been crucial in furthering the understanding of the pathophysiology of PAH more broadly. While a large majority of cases of hereditary PAH are associated with mutations in the bone morphogenic protein receptor type II (*BMPR2*), of particular interest in this review are several recent studies implicating mutations in genes encoding for two different potassium channels—potassium channel subfamily K member 3 (*KCNK3*) and ATP-binding cassette subfamily C member 8 (*ABCC8*)—as PAH-predisposing genes [11,12]. While as of yet, *KCNK3* and *ABCC8* mutations are the only channelopathies known to cause PAH, there is growing data supporting the significant role that potassium channels play in the cellular mechanisms underlying abnormal PAEC and PASMC behavior [1]. Potassium channels therefore represent a compelling target for potential therapies for PAH.

This review focuses on three major potassium channels (or channel subunits) found in the pulmonary vasculature and/or in association with PH, which are divided by their electrophysiological properties and structures: two-pore-domain channels (K2P); the ATP-sensitive potassium channel, a type of inwardly-rectifying potassium channel (Kir); and voltage-gated potassium channels (Kv). We briefly review the molecular biology and mechanism of each channel and the role of each channel in the pulmonary vasculature in the pathophysiology of disease. We discuss PAH-disposing mutations for each channel and how perturbations in each channel or channel subunit lead to PAH. Finally, special attention is placed on potential targets for disease-modifying therapies for PAH. In this sub-section, we detail known inhibitors and activators of the channels of interest, how selective these are for the relevant channel, and what data are still needed to turn these into potential disease-modifying therapeutics.

## 2. KCNK3

### 2.1. Introduction and Molecular Biology

Potassium channels with two-pore alpha subunits were first identified and described in yeast in 1995 [13], and a human homologue was discovered the following year [14]. Two alpha subunits with the two-pore-domain structure come together as homodimers to form the function two-pore-domain potassium channel [15]. The KCNK3 two-pore-domain potassium channel has multiple valid names. It may be referred to as TASK-1 (TWIK-related acid-sensitive K^+^-1, or K2P3.1. In this review, we refer to the channel as KCNK3 throughout.

**Figure 1:**
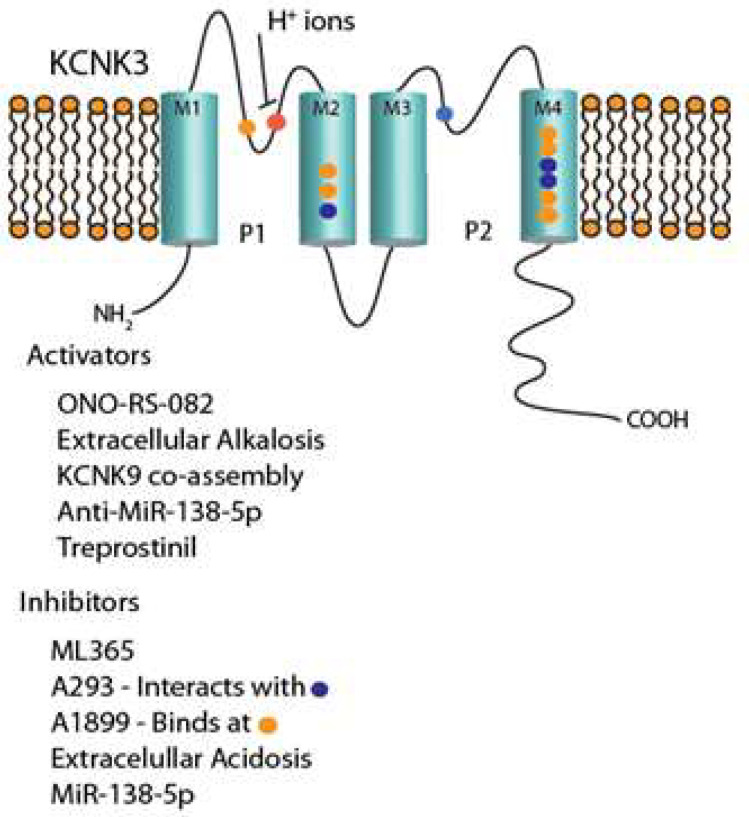
Simplified schematic representation of KCNK3 channel; inhibitors and activators of the channel listed with color-coded regulatory sites. M1–M4 = transmembrane segment number. P1 and P2 = pore domain number. Blue dot = amino acid interaction site of A293. Orange dot = amino acid interaction site of A1899.

The KCNK3 channel is composed of two alpha subunits that each have two pore domains and four transmembrane segments (Figure 1). The two alpha subunits co-assemble to form the functional KCNK3 channel. KCNK3 is considered a “background” K^+^ channel that is non-inactivating and thus would help set the resting membrane potential when expressed on the plasma membrane of a cell. KCNK3 is exquisitely sensitive to extracellular pH, especially near physiologic pH of 7.4. Extracellular protons inhibit channel function when H^+^ ions bind to amino acid residue H98 in the P1 loop (marked by a red dot in Figure 1), while alkalosis activates channel function. Over the last two decades since their discovery in 1995, KCNK3 channels have been shown to play a significant role in modulating electrophysiologic activity in the pulmonary vasculature, to be players in response to hypoxia in the lungs, and to predispose to PH when defective [13,15,16,17].

### 2.2. KCNK3 Channel in PH

In 2002, Gurney et al. described a current IKN, a non-inactivating background K^+^ current, in pulmonary arteries [18]. A year later in 2003, Gurney et al. showed that IKN was carried by KCNK3 in pulmonary myocytes that were derived from rabbit pulmonary arterioles [19]. In 2006, Olschewski et al. used human PASMCs isolated from human lungs and cultured these cells [16]; KCNK3 expression was modulated by an engineered KCNK3 inhibitory siRNA that led to channel knockdown, while additional experiments employed anandamide (a putative KCNK3 inhibitor), acidosis, and hypoxia—as prior studies had—to block channel function. While cultured PASMCs do not seem to maintain the same electrophysiologic characteristics as freshly isolated cells, Olschewski et al. were able to show that KCNK3 is expressed in human PASMCs; that KCNK3 is sensitive to hypoxia and contributes to the resting membrane potential of human PASMCs; and that KCNK3 was activated by the prostacyclin analogue treprostinil at clinically relevant concentrations via protein kinase A [16]. This important study verified a role for KCNK3 in modulating the electrophysiologic profile of PASMCs, including in the setting of hypoxia.

In 2004, Gardener et al. discovered that these channels contribute to regulation of rat mesenteric and pulmonary artery basal membrane potential and tone [20], an important advancement that conveyed the functional significance of two-pore-domain channels as regulators of pulmonary arterial constriction at the organ level.

### 2.3. KCNK3 Mutations in PAH

In 2013, Ma et al. published a landmark paper describing the discovery of KCNK3 as a novel channelopathy in PAH [11]. Using whole-exome sequencing, patients with idiopathic or familial PAH were found to have heterozygous missense variants in the *KCNK3* gene, without identifiable mutations in other genes known to be associated with PAH (e.g., *BMPR2*). A total of six heterozygous mutations were found in six independent patients with idiopathic or familial PAH. All six *KCNK3* variants were predicted to be damaging using in silico bioinformatics. Using whole-cell patch-clamp electrophysiology in COS-7 cells transfected with KCNK3 WT versus mutated channel harboring one of the six PAH-associated mutations, it was found that all six *KCNK3* variants led to loss-of-channel function compared to WT channel, as measured by reduced potassium channel current density. Furthermore, when applying an experimental compound, the phospholipase A2 inhibitor ONO-RS-O82, to cells expressing mutant KCNK3 channel, recovery of some potassium channel current was observed. The degree of loss of channel function, and of recovery of function, varied based on the KCNK3 mutation.

In a follow-up study under heterozygous conditions (e.g., one mutant and one WT channel) in a similar heterologous COS-7 cell expression system, indeed the severity of channel dysfunction varied based on the mutation, and the underlying mechanism of channel dysfunction varied as well (for e.g., V221L mutation demonstrated a change in its pH sensitivity, rendering the KCNK3 channel largely inhibited at physiological pH). Moreover, the degree of recovery of channel function by ONO-RS-082 varied by KCNK3 mutation as well [21]. Other studies since the initial finding by Ma et al. have reported the identification of additional KCNK3 mutations in association with patients with pulmonary arterial hypertension [22,23,24]. 

### 2.4. KCNK3 Dysfunction in Human and Animal Models of PH

In 2016, Antigny et al. reported the physiologic and hemodynamic consequence of KCNK3 loss of function on the pulmonary arterial system in a rat model [25]. Using a monocrotaline-induced PH rat as the experimental substrate, they demonstrated reduction in KCNK3 expression and function. They also saw reduced KCNK3 expression and function in human PAH. The human PAH model was derived from human lung tissue specimens obtained at time of lung transplant from 11 patients with PAH. These samples were compared to 12 control lung samples taken from patients with localized lung cancer at the time of lobectomy or pneumonectomy, and pulmonary artery samples from these controls were taken from sites distant from the known tumor area.

Using patch-clamp electrophysiology of freshly isolated PASMCs and PAECs, Antigny and colleagues demonstrated reduction in KCNK3 current that progressed alongside the development of PH in the monocrotaline rat model, and the reduced KCNK3 current was associated with plasma membrane depolarization [25]. Furthermore, reduction in KCNK3 current at the cellular level was associated with progression toward PH at the tissue level: there was evidence of increased proliferation of PASMCs, PAECs, and adventitial fibroblasts; an increased inflammatory signature was also observed in the setting of prolonged KCNK3 inhibition (28 days), evidenced by increased mRNA levels of inflammatory modulators such as interleukin-17Ra and alpha-1-antitrypsin. These cellular and tissue alterations lead to an overall increase in distal neomuscularization and hemodynamic signs of PH. Western blot and quantitative polymerase chain reaction (PCR) experiments demonstrated that KCNK3 was significantly reduced in PH rats in both PASMCs and PAECs and that KCNK3 channels accumulated in the endoplasmic reticulum in PASMCs while being absent on the plasma membrane.

Altogether, the authors showed that loss of KCNK3 expression and function via reduced KCNK3 currents led to increased pulmonary vascular cell proliferation and inflammation, leading to enhanced pulmonary arterial constriction—pathogenic hallmarks of PH. The authors concluded that KCNK3 loss of function represents a critical cellular event in the pathogenesis of PH (and PAH in humans) and may be an amenable therapeutic target. Furthermore, these results highlight that KCNK3 dysfunction is a hallmark of both heritable and non-heritable causes of PAH, either directly via genetic mutation or indirectly via cellular modulation of ion channel function [25]. Further experimental details of this important study are discussed below.

### 2.5. Cellular Electrophysiology and Pharmacology of KCNK3 PH Rats

Using a series of inhibitors of voltage-gated and ATP-sensitive K^+^ channels, Antigny et al. isolated the current attributable to KCNK3 in isolated rat PASMCs and showed that the current is exquisitely pH-sensitive, a hallmark property of the KCNK3 channel [25]. This suggested functional expression of KCNK3 in freshly isolated rat PASMCs. Notably, over time in the PH rats, there was a progressive decrease in the acid-sensitive current, suggesting that development of PH in rats over time led to a corresponding progressive downregulation of functional KCNK3 expression in PASMCs. For instance, by day 7 after exposure to monocrotaline, the acid-sensitive K^+^ current was reduced by 30%; by day 14, a reduction of 80% and, by day 21, a 90% reduction were observed. The progressive reduction in the acid-sensitive K^+^ current over time in PH rats correlated with an 80% reduction in KCNK3 protein in the lung at day 14 and a 90% reduction at day 21. However, the KCNK3 protein expression was unchanged at day 7 of monocrotaline exposure, despite the fact that the acid-sensitive K^+^ current was reduced by 30% by day 7. This suggests that the acid-sensitive K^+^ current highly likely includes KCNK3, though it is not specific to KCNK3 current alone.

Using current-clamp electrophysiology under whole-cell conditions, Antigny et al. demonstrated progressive resting plasma membrane depolarization, with a plateau of resting membrane depolarization by the 14-day mark. Further reduction in KCNK3 expression beyond 14 days did not lead to further membrane depolarization, suggesting other factors (e.g., ion channels), unsurprisingly, clearly play a role in setting the resting potential in the monocrotaline-induced PH rat model [25]. Indeed, it is important to note that the authors also observed strong reduction in other K^+^ currents and decreased mRNA expression of several voltage-gated potassium channels (e.g., Kv1.2, Kv1.4, Kv2.1, Kv4.2, Kv4.3) in severe PH rats. Experiments in PASMCs were followed by experiments in freshly isolated PAECs. Immunostaining confirmed plasma-membrane localization of KCNK3. After only 7 days in the PH rat model, the acid-sensitive K^+^ current was reduced by 80%, and current-clamp studies showed plasma membrane depolarization to −18.7 mV from −26.6 mV in controls [25].

As extracellular pH is a non-specific K+ channel regulator, and as acidity is known to regulate KCNK3 as well as other K2P channel family members, Antigny et al. used A293, reported as a KCNK3 selective blocker [25]. At high doses of A293, the drug likely has nonspecific effects. Thus, the authors used a moderate dose concentration of A293 (200 nmol/L) and observed a 30% reduction in K^+^ current after its application. In the PH rat model, however, the A293-sensitive current was reduced by 90% in PASMCs, and after applying Kv and KATP channel inhibitors, A293 still reduced K^+^ currents to a similar degree as acidic pH. Furthermore, the resting membrane potential in PASMCs was depolarized to −30 mV from a baseline of −40 mV after addition of A293. Altogether, these data suggest that KCNK3 downregulation in PASMCs leads to decreased whole-cell K^+^ currents and a depolarized resting membrane potential that can be reasonably attributed, at least in part, to reduced KCNK3 currents. Moreover, the decrease in KCNK3 currents can be produced by the development of PH in the monocrotaline rat model [25].

### 2.6. KCNK3 Currents and Pulmonary Arterial Constriction

To assess whether the observed membrane depolarization of PASMCs and PAECs led to pulmonary arterial constriction secondary to reduced KCNK3 expression, pulmonary arteries were isolated and cultured in organ baths and membrane potentials measured [25]. KCNK3 inhibition with the small molecule inhibitor A293 did not lead to contraction of isolated pulmonary arteries, despite high dose concentrations (peak 3 umol/L). This suggested that the membrane depolarization that might be attributable to KCNK3 inhibition alone is not sufficient to depolarize the plasma membrane enough to initiate activation and channel opening of L-type Ca^2+^ channels; thus, no pulmonary arterial vasoconstriction was observed. However, in a follow-up experiment, Antigny et al. pretreated the isolated PAs with Bay-K8644, a drug that potentiates the voltage sensitivity of L-type Ca^2+^ channels. When applying A293 to Bay-K8644 pre-treated PASMCs, significant PA constriction was observed. A293 was also shown to potentiate the vasoconstrictive effect of a thromboxane A2 mimetic U46619. While the signaling pathways involved in KCNK3-mediated pulmonary artery constriction are not fully elucidated, the authors were able to conclude that, at minimum, KCNK3 inhibition by A293 predisposes isolated pulmonary arteries to vasoconstrict [25].

### 2.7. KCNK3 Dysfunction in PH Using a CRISPR/Cas9 Rat

Lambert et al. published in 2019 on an engineered CRISPR/Cas9 KCNK3 knockout rat which recapitulated a PH phenotype [26]. Rat was used over mouse, as KCNK3 in mice does not form a functional channel in PASMCs, while the KCNK6 channel does, and thus mouse is not an appropriate model animal for KCNK3 in PH [27,28]. Thus, Lambert et al. created a 94 bp out-of-frame deletion in exon 1 of the *KCNK3* gene. Patch-clamp electrophysiology showed absence of KCNK3 current from freshly isolated PASMCs from the CRISPR/Cas9 KCNK3 knockout rats, with corresponding resting PASMC plasma membrane depolarization.

At 4 months of age, closed-chest right heart catheterization was performed on the KCNK3-mutated rats. A small increase in right ventricular systolic pressure was observed compared with wild-type (WT) rats, without corresponding RV hypertrophy or change in cardiac output. There was, however, a significant increase in total pulmonary resistance compared to WT rats. Echocardiography at age 4 months showed increased pulmonary artery acceleration time corresponding to higher PA resistance, in KCNK3-mutated rats, along with increased heart rate without changes in other measured echocardiographic parameters. At 12 months old, a significant percentage of the KCNK3 knockout rats demonstrated PH by measure of an elevated RVSP >40 mmHg (45% in males, 36% in females); however, the elevated RVSP was again not associated with RV hypertrophy or a reduced cardiac output [26].

Notably, when exposed to monocrotaline or chronic hypoxia, the KCNK3 knockout rats were sensitized to develop more severe PH, as evidenced by increased RVSP and total pulmonary resistance. This suggests that KCNK3 loss of function and/or downregulation is likely part of the molecular signature underlying PH in general, when induced by either monocrotaline or hypoxia [26].

At the tissue and cellular level, KCNK3 dysfunction led to distal pulmonary vessel neomuscularization, associated with increased collagen crosslinking, and in a corresponding in vitro assay, KCNK3 knockdown or channel inhibition via pharmacologic means (e.g., via siRNA or A293 exposure) increased human PASMC proliferation. KCNK3 knockdown in human PASMCs was associated with increased HIF1-alpha expression and overactivity of the ERK1/2 pathway. At the molecular level, KCNK3 mutation in rats was associated with overactivity of proliferative pathways and altered endothelial cell marker expression. As it relates to endothelial cells, von Willebrand factor (vWF) expression was greatly increased, and CD31 (a marker at endothelial cell-cell junctions) was decreased in the lungs of KCNK3-mutated rats. Moreover, TWIST1, a regulator of the endothelial–mesenchymal transition, was overexpressed; these data together suggest that migrating endothelial cells and a transition to a smooth muscle cell phenotype in the pulmonary vasculature may contribute to KCNK3-mutation-mediated PH [26,29,30,31,32].

At the pulmonary arteriole level, KCNK3 dysfunction predisposed to PA constriction while reducing PA relaxation. In contrast, KCNK3 did not alter vessel constriction or relaxation of rat aorta or pulmonary veins, again suggesting that KCNK3 has specific vasoconstrictive modulatory effects in the pulmonary arterial bed [26].

Overall, the CRISPR-Cas9 KCNK3 knockout rat recapitulated some but not all aspects of a PH phenotype, while demonstrating that KCNK3 dysfunction is strongly associated with the development of PH in general, supported by molecular-, electrophysiologic-, and tissue-level alterations in the pulmonary vasculature [26].

### 2.8. Signaling Pathways Involved in KCNK3 Regulation

Several intracellular signaling pathways have been described that affect KCNK3 channel activity and thus might be exploited for therapeutic purposes in PH. These include studies involving microRNAs that may contribute to hypoxia-associated PH via KCNK3 channel regulation [33,34].

For instance, the microRNA MiR-138-5p is predicted to regulate KCNK3 expression. In a monocrotaline-induced PH rat model, anti-MiR-138-5p, delivered via nebulizer to the rats, led to reduced right ventricular systolic pressure and improved pulmonary artery acceleration time, and in vivo inhibition of MiR-138-5p led to restoration of KCNK3 mRNA expression, suggesting that inhibition of MiR-138-5p reduces the PH phenotype in monocrotaline-induced PH rats at least in part via recovery of KCNK3 expression [33].

It was demonstrated that resistin-like molecule B (RELM-B), a protein involved in pulmonary disease states including pulmonary inflammation, fibrosis, and asthma had significantly increased protein levels under chronic hypoxia in human PASMCs, while KCNK3 protein expression decreased. RELM-B led to human PASMC proliferation and activation of the STAT3/NFAT signaling pathway via KCNK3 downregulation under normoxic conditions, while inhibiting RELM-B expression interfered with KCNK3-mediated PASMC proliferation under hypoxic conditions. Phospholipase C (PLC) was implicated in this interaction, as PLC inhibition prevented decreased KCNK3 protein expression in the setting of hypoxia/RELM-B and in turn inhibited PASMC proliferative potential. Altogether, these results suggest that RELM-B activation is involved in hypoxia-induced PASMC proliferation as is KCNK3 inhibition and that RELM-B inhibition may lead to PLC-dependent KCNK3 activity that may reduce hypoxia-associated PH [35].

Using proteomic analysis, it was demonstrated that loss of expression of KCNK3 in human PASMCs and PAECs leads to differential protein expression. Proteomic remodeling in PASMCs included activation of the PI3K/AKT pathways and reduction in EIF2 signaling and purine nucleotide de novo biosynthesis II and IL-8 signaling pathways; proteome remodeling in PAECs was mostly associated with activation of mTOR signaling, glycolysis, and the superpathway of methionine degradation and a reduction in EIF2 signaling. Common to both PASMCs and PAECs, KCNK3 loss of expression led to reduction in the interferon pathway and activation of the NRF2-mediated oxidative stress response. Altogether, the proteome alterations in the setting of KCNK3 dysfunction favored proliferation and migration of PASMCs and apoptosis resistance and a change in metabolism in PAECs [36].

### 2.9. KCNK3 and Immune Cells in PAH

Recent studies implicate altered immune response as an overlapping phenotype between PAH-patient-derived cells and a mouse KCNK3 knockout model, suggesting that alterations in immune cells may underlie susceptibility to PAH in patients with KCNK3 mutation [37]. Moreover, peripheral recirculating immune cells between healthy controls and KCNK3 hereditary PAH patients show significant differences, including a relative reduction in naïve CD8+ and CD4+ T cells and naïve B cells, and reciprocal increases in memory CD8+ and CD4+ T cells and double negative CD4−/CD8− T cells. The authors of this study conclude that KCNK3 mutation may predispose to increased inflammation via multiple intrinsic pathways [37]. This area of research highlights additional potential points of intervention in KCNK3-mediated PAH beyond the KCNK3 channel complex itself.

### 2.10. KCNK3 Inhibitors and Activators

As KCNK3 dysfunction can play a significant role in the pathogenesis of PAH, there is great interest in developing selective pharmacologic agents to block and activate the channel, for experimental and therapeutic purposes, respectively (Figure 1). A variety of KCNK3 inhibitors have been described, none fully selective, though with varying degrees of selectivity. The following section highlights some inhibitors and activators of KCNK3.

### 2.11. KCNK3 Inhibitors

#### 2.11.1. A293

A293 is an aromatic carbonamide developed by Sanofi, which at 1 uM concentration in a heterologous expression system inhibits 75% of KCNK3 and 50% of KCNK9 channel current density [38], with minimal inhibition of other channels tested, including some voltage-gated and two-pore-domain potassium channels. Using alanine-scanning mutagenesis, Wiedmann et al. discovered Q126, located in the M2 segment of the channel, along with L239 and N240 located in the M4 segment, as the amino acids necessary for A293 mediated KCNK3 inhibition. In silico docking experiments demonstrated a proposed A293 binding site in the central cavity of KCNK3, at the low end of the pore on the cytosolic side, at the entry to lateral side fenestrations of KCNK3 [39].

#### 2.11.2. A1899

A1899 is a compound shown to inhibit KCNK3 with 90% efficacy and KCNK9 with 20% efficacy, at 100 nM concentration, with minimal inhibition of other potassium channels tested (including voltage-gated and two-pore-domain channels) [40]. A1899 serves as an open-channel blocker, binding to amino acid residues within the central cavity. Residues in both pore loops, the M2 and M4 transmembrane segments, and the halothane response element were noted to form the A1899 drug binding site of KCNK3 after alanine mutagenesis screening and further supported an open-pore homology model of KCNK3 [40].

#### 2.11.3. ML365

ML365, a bis-amide-derived compound, is perhaps the most selective small molecule compound against KCNK3. It is commercially available and has been shown to confer 62-fold selectivity of KCNK3 over the closely related KCNK9, with an IC50 = 16 nM [41]. Experiments in cultured human PASMCs heterologously expressing transfected KCNK3 tagged to a green-fluorescent protein (GFP) marker, demonstrate that ML365 virtually fully inhibits KCNK3 currents produced by the transfected KCNK3-GFP construct, and inhibition of overexpressed KCNK3 led to cultured human PASMC plasma membrane depolarization [21].

### 2.12. KCNK3 Activators

#### 2.12.1. ONO-RS-082

Described as a phospholipase A2 inhibitor as its primary mechanism of action, ONO-RS-082′s mechanism of action on KCNK3 channels remains unknown. The rationale for using ONO-RS-082 as a modulator of KCNK3 channel activity came after a patent through Columbia University to explore phosphorylation of the KCNK3 channel yielded ONO-RS-082 as a small molecule compound that was associated with KCNK3 regulation [4]. ONO-RS-082, a small molecule inhibitor, has been shown to enhance KCNK3 current in heterologous expression systems using cultured cell lines [11,21]. Studies showed that ONO-RS-082, while non-specific for KCNK3 current, may activate wild-type KCNK3 channel in addition to some mutated channels associated with PAH and that activation of KCNK3 by ONO-RS-082 may occur in the presence of homodimeric (e.g., only WT channel subunits) or heterodimeric (e.g., one WT subunit plus one mutant subunit) channels [11,21]. Notably, not all mutant KCNK3 channels associated with PAH show recovery of function in the presence of ONO-RS-082 [11,21,42].

In a monocrotaline-induced rat model of PH, treatment of rats with ONO-RS-082 at day 1 of monocrotaline exposure, and successively through day 21 with an injection of 50 mg/kg of ONO-RS-082 per day, led to reduction in the severity of PH. This reduction was surrogately measured by a 50% reduction in right ventricular systolic pressure and a 60% reduction in right ventricular hypertrophy, as well as a reduction in vascular wall thickness, compared to monocrotaline-induced PH rats without exposure to ONO-RS-082 [25].

Interestingly, while monocrotaline-PH rats not exposed to ONO-RS-082 showed decreased acidic-K^+^ sensitive current (ie. KCNK3 current) at 14 days (80% reduction) and 21 days (90% reduction) post-monocrotaline exposure, as well as decreased lung KCNK3 protein expression of 80% to 90% at 14 and 21 days post-monocrotaline exposure, the monocrotaline-exposed rats that were simultaneously treated with ONO-RS-082 from days 1 through 21 of monocrotaline exposure showed lung KCNK3 expression similar to controls by Western blot analysis [25]. Altogether, this suggests that ONO-RS-082′s mechanism of action on KCNK3 may relate to increased channel expression and/or stabilization of KCNK3 channels at the plasma membrane and/or decreased channel degradation, leading to increased KCNK3 activity and current density. This hypothesis warrants further investigation.

#### 2.12.2. KCNK9

KCNK9, also named TASK-3, is a related two-pore-domain potassium channel with a shifted acid–base channel sensitivity profile, rendering KCNK9 more maximally activated at pH 7.4 than KCNK3 [21,38,43]. KCNK9 is 62% identical to KCNK3, with high levels of expression in the central nervous system [43]. KCNK9 is expressed in some tissues outside of the central nervous system as well [44,45,46,47]. As it relates to KCNK3 and its association with PAH, it was shown that KCNK3, but not KCNK9, is expressed in healthy and PAH patient lungs [21].

KCNK3 and KCNK9 can coassemble, forming functional channels from heterodimers consisting of one KCNK3 subunit and KCNK9 subunit [45,46,47,48]. After engineering a tandem-linked construct of human KCNK3-KCNK9 tandem-linked subunits, it was shown that the KCNK3-KCNK9 heterodimeric channels demonstrate increased K^+^ current activity at physiologic pH 7.4, compared to KCNK3 homodimeric channels [21]. Furthermore, KCNK9 subunits heterodimerized to a mutant KCNK3 subunit associated with PAH (e.g., G203D KCNK3) demonstrated increased K^+^ current activity compared to G203D KCNK3 associated with WT KCNK3 channel. This supports the notion that KCNK9 can coassemble with mutant KCNK3 and produce functional recovery at physiologic pH in a heterologous expression system, providing protection against KCNK3 dysfunction. By extension, as KCNK9 and KCNK3 are co-expressed in a variety of tissues outside the lungs, it was proposed that perhaps the *absence* of KCNK9 in the lungs may underlie the lung-specific phenotype observed in PAH patients due to KCNK3 mutation, and KCNK9 gene therapy may represent a novel therapeutic approach to PAH due to KCNK3 dysfunction [21].

## 3. ABCC8/SUR1

### 3.1. Introduction and Molecular Biology

**Figure 2:**
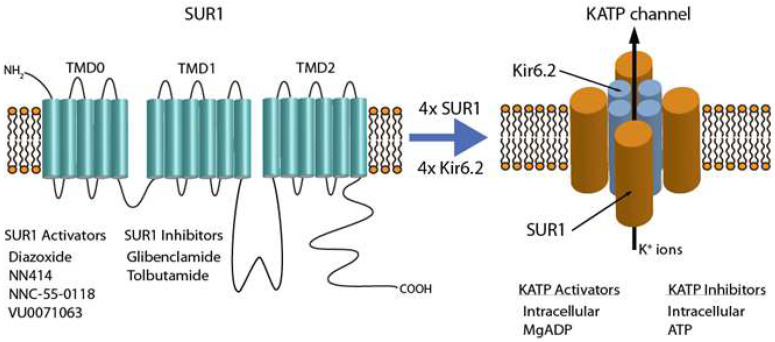
Simplified schematic representation of the SUR1 subunit and KATP channel; key inhibitors and activators of SUR1 listed.

ATP-sensitive potassium channels (KATP) help control a variety of physiologic processes in the human body, including insulin secretion from pancreatic beta cells and cardiovascular cell excitability, as they act as molecular sensors and thus regulators of cell metabolism [49,50]. KATP channels provide a link between cell metabolism and cell excitability [51]. KATP channels are composed of a hetero-octamer of four pore-forming subunits and four regulatory subunits (Figure 2). More specifically, the pore-forming inwardly-rectifying potassium channel subunit, or Kir6.x subunit (Kir6.1 or Kir6.2), combines with a sulfonylurea receptor (SUR) subunit [52], namely SUR1 or SUR2, with two splice variants of SUR2 named SUR2A and SUR2B. In a 1:1 stoichiometric ratio, four Kir6.x subunits combine with 4 SUR.x subunits to form the functional KATP channel. Both Kir6.x subunits and SUR regulatory subunits are required for channel activity [53].

KATP channels are inhibited by intracellular ATP, mediated by Mg^2+^ ions, and activated by MgADP or the dissociation of intracellular ATP from the channel. SUR1 may be described as an “ADP sensor” [54].

ATP Binding Cassette Subfamily C Member 8 (*ABCC8*) is the gene that encodes SUR1 [51]. *ABCC8* is located on chromosome 11 along with *KCNJ11*, the gene encoding Kir6.2, while *ABCC9*, the gene encoding SUR2, is located on chromosome 12, along with *KCNJ8*, the gene encoding Kir 6.1 [50]. The expression of KATP channels occurs in different compositions depending on tissue type. SUR1 is highly expressed in neurons and the pancreas [49,50], and SUR2 expression is the predominant SUR isoform in endothelial and smooth muscle cells [55].

With regards to predominant KATP subunit composition in the cardiovascular system, the atrium comprises KATP channels from Kir6.2 and SUR1 and SUR2A subunits [56,57], while in the ventricle, KATP is composed of Kir6.2 and SUR2A subunits [57,58]. The conduction system and endothelial cells express Kir6.1, Kir6.2, and SUR2B [59,60], while vascular smooth muscle cells express Kir6.1 and SUR2B predominantly [50].

### 3.2. SUR1/KATP Channels in PAH

In 2018, the association between *ABCC8*/SUR1 loss-of-function mutations and patients with PAH was first reported [12]. Using exome sequencing and two different PAH patient cohorts, 12 different heterozygous predicted deleterious novel and rare variants in *ABCC8* were identified. Whole-cell patch-clamp analysis and rubidium flux assays demonstrated that all 11 *ABCC8* variants led to loss of KATP channel function when co-expressed heterologously with Kir6.2. One *ABCC8* variant of the two could not be functionally studied as it led to no functional protein. Importantly, rescue of all mutant channels (SUR1 mutant plus Kir6.2 in vitro) was observed upon application of diazoxide, a selective SUR1 activator [12].

The SUR1 mutations associated with PAH occurred at amino acid residues that were highly conserved across species. The degree of loss of KATP channel currents when combining mutant SUR1 and Kir6.2 heterologously varied based on mutation, and the degree of recovery of channel function varied as well after the application of diazoxide. To ensure that the KATP channel activation was due to recovery of function of SUR1 specifically, as opposed to augmenting function of Kir6.2 alone, glibenclamide, a SUR-specific KATP channel inhibitor was applied after diazoxide application in whole-cell patch-clamp studies. The glibenclamide-sensitive current was taken as the SUR1 dependent current, and this SUR1 dependent current was increased for all 11 mutant SUR1 associated with PH after application of diazoxide. It was therefore concluded that loss of function mutations of SUR1, a regulatory subunit of KATP channels, is associated with PAH and that channel function can be rescued via pharmacologic activation; thus, SUR1 and KATP channels may represent a therapeutic target in PAH [12].

The finding of *ABCC8* variants associated with PAH was corroborated in a Spanish PAH registry, where predicted pathogenic *ABCC8* variants were found associated with PAH using in silico tools coupled to biochemical studies [61].

### 3.3. SUR1 Loss-of-Function in PAH

Interestingly, while SUR1 loss of function is an established cause of congenital hyperinsulinism, none of the patients in the study identifying loss of SUR1 function in PAH had evidence of hyperinsulinemic hypoglycemia or transient/permanent neonatal diabetes mellitus. The reasons for the variable expressivity of loss of SUR1 function remain unknown, though it is likely due to a combination of genetic, developmental, and environmental factors. Moreover, while diazoxide is a mainstay treatment in congenital hyperinsulinism secondary to SUR1 loss of function, hypoglycemic infants treated with diazoxide have actually developed PH [62], while case reports from decades ago show that diazoxide can reverse PH [63,64]. Furthermore, four missense loss of function SUR1 mutations have been identified in patients with either PAH or congenital hyperinsulinism (CHI), without overlap of these clinical syndromes, and four variants are associated with both PAH and CHI, suggesting variable penetrance and/or expressivity in the variety of tissue types that rely on SUR1 function [12].

Diazoxide as a pharmacologic therapy in PH may introduce too much toxicity and was thus not recommended based on the available data. Its experimental use in association with SUR1 mutations in PAH, however, has provided proof of concept that the pharmacologic rescue of mutant SUR1 is plausible and that SUR1-containing KATP channels with PAH-associated mutations are a potential druggable target in PAH [12].

The mechanism of how SUR1 loss of function leads to PAH remains elusive [55]. SUR2 (encoded by ABCC9) most likely serves as the predominant SUR isoform expressed in human lungs [55,65]. SUR1 expression in intact rat pulmonary arteries was observed in addition to SUR2B and Kir6.1. Human pulmonary arteries expressed only SUR2B and Kir6.1 in the same study [65]. Notably, the expression in human tissue was derived from cultured PASMCs, which may not retain the same expression profile after culturing. Indeed, it was more recently demonstrated that *ABCC8* is expressed in lungs of healthy individuals and patients with PAH via reverse transcriptase polymerase chain reaction (RT-PCR) and that SUR1 protein, using immunohistochemistry, exhibited strong staining in alveolar macrophages and moderate staining in proximal pulmonary arteries of lung samples from patients with idiopathic PAH [12]. *ABCC8* expression was also up-regulated in human lung tissue samples from PAH patients with known BMPR2 mutations [12].

### 3.4. SUR1/KATP Channels and Vasoreactivity in the Pulmonary Vasculature

More recently, it was shown that protein expression via Western blot of SUR1 from control patients and patients with idiopathic or heritable (due to BMPR2 mutation) PAH revealed similar levels of expression in lung tissue samples and that protein expression from isolated pulmonary arteries was not different either [66]. A similar pattern of expression was observed for Kir6.2. Furthermore, immunohistochemistry revealed colocalization of SUR1 and Kir6.2 in human PAECs and PASMCs. After application of the SUR1 activator, diazoxide, the proliferation rate of control human PAECs and PASMCs was reduced without changing proliferation rates of PAECs or PASMCs from human idiopathic PAH samples [66]. To test SUR1’s contribution to pulmonary arterial vasoreactivity, pulmonary artery samples were precontracted with U46619 (a thromboxane A2 mimetic), and diazoxide was applied, which led to PA relaxation by 16.5% compared with PA treated with DMSO alone. Pharmacologic activation of SUR1 induced PA relaxation in samples taken from monocrotaline-induced PH rats and in human idiopathic PAH patient samples. The degree of PA relaxation induced by diazoxide was similar in idiopathic PAH PA samples as compared to control PA samples [66].

Perhaps SUR1 serves a regulator role to mitigate against the development of PH when functioning normally. Taken together, SUR1 as a potential therapeutic target in human lungs in patients with PAH is plausible; however, the molecular mechanism, cell type, and signaling pathways involved remain unclear.

SUR1 activity in the lung that does not involve KATP channel activity is possible. SUR1 has been reported to coassemble with the nonselective pore-forming channel subunits TRPM4 to form SUR1-TRPM4 channel complexes in the nervous system [55,67]. TRPM4 is also expressed in vascular and pulmonary smooth muscle [12,68,69]. As the possibility for TRPM4-SUR1 coassembly has been scrutinized [70], and as no known study has reported its function in the lung, any involvement of SUR1-TRPM4 channels in PAH pathology remains speculative and without an obvious mechanism.

Interestingly, in vitro studies show that SUR1 can mediate apoptosis via a KATP-channel-independent mechanism [71,72]. If this theory can be extrapolated to the human lung and SUR1 expression already established in lung macrophages and pulmonary arteries [12], perhaps SUR1 loss of function leads to decreased apoptosis and enhanced vascular hyperplasia and/or inflammation which predisposes to the PH phenotype. This hypothesis warrants further investigation.

### 3.5. Inhibitors of SUR1/KATP Channel Function

Inhibition of KATP channels by intracellular ATP is a defining property of these ion channels. More specifically, the open probability of the KATP channel greatly decreases in the presence of cytosolic ATP [50]. Nonhydrolyzable ATP analogs, or the absence of Mg^2+^, can also inhibit channel activity [50,73]. Free ADP is also an inhibitor of KATP channels, though with much less potency than ATP. Ultimately, it was established that the physiologic regulation of KATP channel opening is dependent on the ATP/ADP ratio as opposed to the concentration of ATP alone [50,74].

The most frequently employed KATP channel inhibitors include the sulfonylureas such as glibenclamide and tolbutamide [50,75]. While falling out of favor clinically due to their adverse side effect profile (e.g., hypoglycemia), sulfonylureas such as glibenclamide have been used clinically for treatment of patients with type II diabetes mellitus. By inhibiting KATP channel function formed by SUR1 and Kir6.2 subunits in the pancreatic beta cells, sulfonylureas enhance insulin release. Glibenclamide exhibits high-affinity binding to SUR1, with a *K_d_* of approximately 0.5–10 nM for glibenclamide binding [50,76,77].

### 3.6. Activators of SUR1/KATP Channel Function

A variety of SUR1/Kir6.x KATP channel activators have been experimentally employed, including pinacidil at >1 mM, diazoxide at 10 uM, and HMR-1098 at 3–33 uM or 860 uM [50]. Diazoxide is a SUR-dependent activator of KATP channel function, strongly activating channels containing the SUR1 isoform (Figure 2) [50]. Diazoxide application in heterologous expression systems can recover KATP channel function containing PAH-associated SUR1 mutations coassembled with Kir6.2 [12] and was more recently shown to attenuate the development of PH in rats exposed to monocrotaline or chronic hypoxia using a protocol of diazoxide exposure of 20 mg/kg/d for 21 days [66].

However, diazoxide has off-target effects in addition to its KATP channel activation; diazoxide can activate SUR2 (not only the SUR1 isoform), and other ion channels or ATPases, and may regulate mitochondrial energetics [66,78]. As such, more selective activators of SUR1 or SUR1/Kir6.2 channels may be clinically beneficial. The experimental compounds VU0071063 and NN414 possess higher selectivity for SUR1/Kir6.2 channels (Figure 2) and may represent preferred pharmacologic agents for treating congenital hyperinsulinism over diazoxide [79]. By extension, both VU0071063 and NN414 were shown to induce PA relaxation and alleviate the PH phenotype in the monocrotaline-induced PH rat model [66]. In summary, SUR1-specific activators warrant further investigation to assess their utility in treating individuals with PAH and PH in general.

## 4. Kv Channels

### 4.1. Introduction and Molecular Biology

Voltage-gated potassium (Kv) channels are major regulators of both cell excitability and resting membrane potential (Em) in vascular smooth muscle cells. Kv channels are constitutively active under resting conditions—thereby maintaining Em—and become further activated by membrane depolarization. During depolarization, Kv channels are especially important in controlling cytosolic calcium concentration ([Ca^2+^]_cyt_) through the inhibition of voltage-gated Ca^2+^ channels on both the plasma membrane and sarcoplasmic reticulum [80,81].

**Figure 3:**
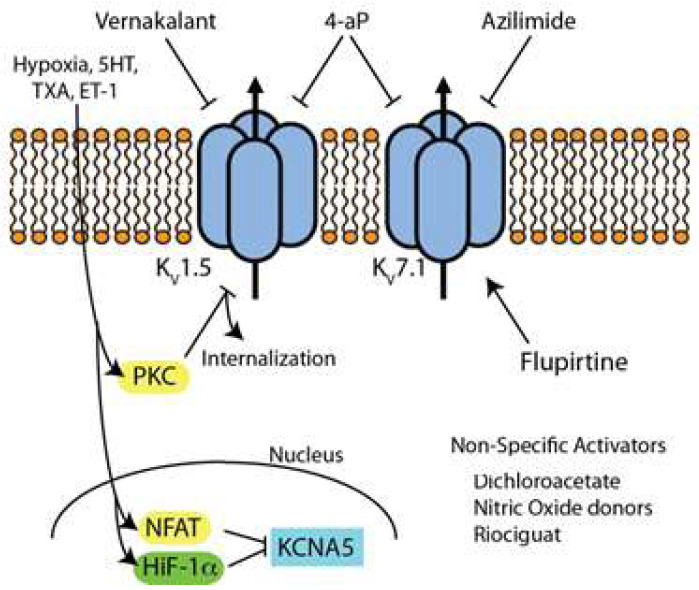
Simplified schematic representation of pathways and small molecules involved in the regulation of the Kv channel; 5HT, serotonin; TXA, thromboxane; ET-1, endothelin-1; PKC, protein kinase C; NFAT, nuclear factor of activated T-cells; HiF-1α, hypoxia-inducible factor-1α, 4-AP, 4-Aminopyridine.

Kv channels form either homo- or heterotetramers of an α-subunit (Kv1–Kv12) with six transmembrane domains per subunit (Figure 3). The six transmembrane domains per subunit possess a voltage-sensing domain and a pore domain. Diversity of Kv channel activity is accentuated by functionally necessary accessory β-subunits which also form homo- or heterotetramers and associate with the α-complex [82].

### 4.2. Kv Channels in PH

Kv channels are expressed in smooth muscle cells throughout the mammalian body. Several types of Kv channels—including Kv1.2, Kv1.5, Kv2.1, Kv3.1, Kv7.1, and Kv9.3—are expressed in PASMCs with regional diversity across the pulmonary vascular bed. Special attention has been placed on the Kv1.5 channel (*KCNA5* gene) because of its regional expression in the small-resistance pulmonary arteries that are particularly important in both normal and disordered function of the pulmonary vasculature [83].

Kv channels play an important role in cell proliferation and cell death in PASMCs. Increased K^+^ current enhances cell death through a conserved mechanism characterized by early apoptotic cell shrinkage. Conversely, decreased K^+^ current inhibits PASMC apoptosis, in part due to the effects of increased [Ca^2+^_cyt_. Highlighting the necessity of increased K^+^ currents in PASMC apoptosis, when rat PASMCs were simultaneously exposed to the pro-apoptotic agent staurosporine and anti-apoptotic agent Bcl-2, BCL inhibited the staurosporine-induced Kv current increase, and PASMCs hypertrophied and proliferated [84,85,86,87].

In addition to their class-wide role as mediators of Em, cell excitability, and resting smooth muscle tone, the Kv1.5 and Kv2.1 channels are key regulators of acute and chronic hypoxic vasoconstriction. The mechanism of both acute and chronic hypoxic vasoconstriction in relation to Kv channels has been well described. In response to acute hypoxia, inhibition of the production of reactive oxygen species inhibits Kv1.5 leading to membrane depolarization, increased [Ca^2+^]_cyt_, and increased PASMC tone. In response to chronic hypoxia, hypoxia-inducible fact 1-alpha (HIF1-α) and nuclear factor of activated T-cells (NFAT) are overexpressed causing reduced expression and function of Kv1.5 and thereby stimulating PASMC hypertrophy and cell proliferation [3,88,89,90].

Many other members of the Kv channel family present in the pulmonary vasculature do not appear to be reactive to hypoxic conditions, consistent with their regional expression in the larger conduit pulmonary arteries which tend not to contribute to hypoxic vasoconstriction [83,91].

Kv channels are sensitive to both the direct effects of hypoxia and indirectly from hypoxia-induced expression of signaling factors. Together these direct and indirect effects lead to dysregulated cell hypertrophy and proliferation, decreased apoptosis, and pulmonary vascular remodeling. As previously reviewed in depth by Boucherat et al. [3], serotonin (5-HT) and thromboxane A2 (TXA)—as well as other endogenous small molecules including endothelin-1 (ET-1) and platelet-derived growth factor (PDGF)—have been shown to negatively regulate Kv expression and function in PAH models. 5-HT appears to modulate Kv current in PASMCs primarily through the activation of phospholipase C leading to increased endocytosis and internalization of the Kv1.5 and Kv2.1 channels [3,92,93,94]. TXA inhibits the Kv1.5 channel via a PKCζ-dependent mechanism (Figure 3) [95].

### 4.3. PAH-Disposing Mutations and Kv Channels

Unlike *KCNK3* and *ABCC8*, there are no known mutations in *KCNA5* associated with hereditary PAH. Nonetheless, given the abundance of research demonstrating the Kv channel’s role in hypoxic vasoconstriction and PASMC apoptosis, proliferation, and remodeling, Kv channels may play an important role in the pathophysiology of PAH and in turn would represent appealing therapeutic targets.

The majority of known PAH-disposing genetic mutations arise in the *BMPR2* gene. A number of studies have looked at the effects of BMPR2 mutations on Kv channel expression and function in both mouse and human models to determine if abnormalities in BMPR2 mutants are mediated by Kv expression and function [96,97,98,99]. Fantozzi et al. found that Kv1.5 expression and current density were significantly reduced in human PASMC BMPR2 mutants, an effect which was reversed with the treatment of recombinant BMPR2. How BMPR2 signaling affects Kv1.5 expression and function is still not fully understood but may be mediated via anti-apoptotic protein Bcl-2 [97].

There are mixed data on the potential role of *KCNA5* single-nucleotide polymorphisms (SNPs) in PAH. Wipff et al. identified a variant SNP in the *KCNA5* promoter region of a European Caucasian cohort which was significantly associated with systemic sclerosis and PAH [100]. Remillard et al. identified multiple SNPs in the 5′ untranslated region (UTR), two SNPs in the translated region, and two SNPs in the 3′ UTR associated with PAH [101]. The exact mechanism of how these SNPs may affect *KCNA5* expression and function is not yet elucidated, though together these data suggest *KCNA5* variants could modulate PAH onset and severity. Complicating these findings, two recent meta-analyses by Jiao et al. and Rhodes et al. found no association between *KCNA5* SNPs and the development of PAH [102,103].

While there is no Kv channelopathy directly associated with heritable PAH, a nonsense mutation in the *KCNA5* gene has been identified in a familial case of atrial fibrillation highlighting the channels’ importance in the mammalian cardiac conduction system [104,105].

### 4.4. Inhibitors/Activators of Kv Channels (Figure 3)

Given the abundance of data associating PAH and Kv current density, Kv may be an ion channel target amenable to disease-modifying therapy. The goal of any Kv-directed therapeutic would be to increase overall Kv current and thereby decrease and reverse PASMC hypertrophy and remodeling of the pulmonary arterial wall. Nonetheless, multiple pharmacological agents have been tested in experimental models to assess their direct effect on Kv channels.

### 4.5. Kv Channel Blockers

The classical non-specific inhibitor of Kv is 4-aminopyridine which has been shown to interact with Kv by binding the channel in an open state and then inducing a bound closed state [106]. Given the abundance of Kv1.5 and Kv7.1 in atrial tissue, a number of specific inhibitors to these channels have been developed as potential anti-arrhythmic agents including multiple which have progressed into clinical trials. Vernakalant is an inhibitor of Kv1.5 that has gained conditional FDA approval for intravenous conversion of atrial fibrillation [107]. Selective blockers of Kv7.1 such as azimilide have been shown to block the slowly activating delayed rectifier potassium current and are under study for efficacy in atrial arrhythmias [107].

### 4.6. Kv Channel Activators/Up-Regulators

To date, there is no direct selective activator of Kv1.5. Niflumic acid was shown to activate Kv7.1 in Xenopus oocytes by stabilizing these channels in the open state. No studies have looked at niflumic acid in PAH models. Another direct activator of Kv7.1, flupirtine, effectively prevented and reversed hypoxia-induced PAH in a mouse model. Flupirtine is a non-opioid analgesic that increases Kv7.1 current by inducing a hyperpolarizing shift of voltage-dependent channel activation and increasing channel open probability [108]. Kv7.1 activators have been suggested as a potential treatment for long QT syndrome [107].

Interestingly, since Kv channels are mainly downregulated via transcriptional and trafficking mechanisms in PAH, the benefit of selective Kv channel openers perhaps is limited [3]. A number of indirect up-regulators, however, have been shown effective in animal models. Dichloroacetate, for example, can prevent and reverse chronic hypoxic PH in rats by restoring expression and function of the Kv1.5 and Kv2.1 channels [109]. Patch-clamp data suggest increased Kv7 current density is a key mechanism underlying the benefits of NO donors and riociguat for PAH treatment [110].

### 4.7. Kv Channel Future Studies

Despite limited success to date with direct small molecule activators of Kv channels for PAH treatment in experimental models, a number of studies involving both post-transcriptional targets and gene therapies show signals of success. Via miRNA expression profiling in PAH-PASMCs and normal PASMCs, miR-29b was found to be a strong post-transcriptional down-regulator of Kv1.5 expression. Inhibition of miR-29b via antisense oligonucleotides rescued Kv1.5 protein levels and restored normal K^+^ current in isolated rat PASMCs [111]. Similarly, transfection of KCNA5 into isolated rat PASMCs increased K^+^ current and enhanced apoptosis [85]. Furthermore, in rats subjected to chronic hypoxia causing decreased Kv currents, in vivo gene transfer of *KCNA5* reduced PH and restored appropriate hypoxic pulmonary vasoconstriction mechanisms [112]. In sum, there are several potential pathways and still much work to be done in order to create a functional therapeutic which targets the Kv channel directly or indirectly in PAH.

## 5. Conclusions

Potassium channelopathies and potassium channel dysfunction are now an established entity that contributes to the molecular mechanisms and pathophysiology leading to PAH. Namely, mutations in the KCNK3 channel, and the *ABCC8* gene that encodes the SUR1 subunit of the KATP channel, may underlie genetically predisposed PAH, and KCNK3 and/or KATP channel dysfunction may contribute to the development of PH in general. Kv channel dysfunction may contribute to PAH as well, though a genetic association of PAH with Kv channel mutations is less established to date.

The degree of contribution of potassium channel dysfunction to both PAH and PH development remains to be discovered. Potassium channel dysfunction underlies vascular cell deleterious remodeling and unwanted proliferation in the pulmonary vasculature in PAH. The therapeutic potential to help patients with PAH and PH remains promising. As a scientific community, we may now be ready to apply the discovery of potassium channel dysfunction as a key pathophysiologic mechanism underlying PAH to develop new druggable targets in PAH, namely, KCNK3, KATP, and Kv channels; to leverage this molecular mechanism in PAH for rational drug design; and ideally, to advance personalized medicine in patients suffering from PAH in the years to come.

## Data Availability

Not applicable.
